# Effects of treadmill exercise versus Flutter® on respiratory flow and sputum properties in adults with cystic fibrosis: a randomised, controlled, cross-over trial

**DOI:** 10.1186/s12890-016-0360-8

**Published:** 2017-01-11

**Authors:** Tiffany J. Dwyer, Rahizan Zainuldin, Evangelia Daviskas, Peter T. P. Bye, Jennifer A. Alison

**Affiliations:** 1 Discipline of Physiotherapy, Faculty of Health Sciences, University of Sydney, Sydney, Australia; 2 Department of Respiratory Medicine, Royal Prince Alfred Hospital, Sydney, Australia; 3 Central Clinical School, Sydney Medical School, University of Sydney, Sydney, Australia; 4 Rehabilitation Department, Ng Teng Fong General Hospital, Jurong Health Services, Jurong East, Singapore; 5 Health and Social Sciences, Academic Programme, Singapore Institute of Technology, Jurong East, Singapore; 6 Department of Physiotherapy, Royal Prince Alfred Hospital, Sydney, Australia

**Keywords:** Cystic fibrosis, Exercise, Oscillating PEP, Flutter®, Airway clearance, Physiotherapy, Sputum

## Abstract

**Background:**

Treadmill exercise and airway clearance with the Flutter® device have previously been shown to improve mucus clearance mechanisms in people with cystic fibrosis (CF) but have not been compared. It is therefore not known if treadmill exercise is an adequate form of airway clearance that could replace established airway clearance techniques, such as the Flutter®. The aim of this study was to evaluate respiratory flow, sputum properties and subjective responses of treadmill exercise and Flutter® therapy, compared to resting breathing (control).

**Methods:**

Twenty-four adults with mild to severe CF lung disease (FEV_1_ 28–86% predicted) completed a three-day randomised, controlled, cross-over study. Interventions consisted of 20 min of resting breathing (control), treadmill exercise at 60% of the participant’s peak oxygen consumption and Flutter® therapy. Respiratory flow was measured during the interventions. Sputum properties (solids content and mechanical impedance) and subjective responses (ease of expectoration and sense of chest congestion) were measured before, immediately after the interventions and after 20 min of recovery.

**Results:**

Treadmill exercise and Flutter® resulted in similar significant increases in peak expiratory flow, but only Flutter® created an expiratory airflow bias (i.e. peak expiratory flow was at least 10% higher than peak inspiratory flow). Treadmill exercise and Flutter® therapy resulted in similar significant reductions in sputum mechanical impedance, but only treadmill exercise caused a transient increase in sputum hydration. Treadmill exercise improved ease of expectoration and Flutter® therapy improved subjective sense of chest congestion.

**Conclusions:**

A single bout of treadmill exercise and Flutter® therapy were equally effective in augmenting mucus clearance mechanisms in adults with CF. Only longer term studies, however, will determine if exercise alone is an adequate form of airway clearance therapy that could replace other airway clearance techniques.

**Trial registration:**

Australian and New Zealand Clinical Trials Registry, Registration number #ACTRN12609000168257, Retrospectively registered (Date submitted to registry 26/2/2009, First participant enrolled 27/2/2009, Date registered 6/4/2009).

**Electronic supplementary material:**

The online version of this article (doi:10.1186/s12890-016-0360-8) contains supplementary material, which is available to authorized users.

## Background

Cystic fibrosis (CF) lung disease is characterised by reduced hydration at the airway surface and dehydrated mucus, [1] resulting in impaired mucus clearance that leads to a cascade of inflammation and progressive lung damage [2]. Interventions to improve mucus clearance are integral to the respiratory management of CF [3].

Most therapies are required daily and adults with CF report spending an average of 108 min on treatment activities each day, the majority of that time performing airway clearance and exercise [4]. Strategies to combine effective interventions to minimise treatment time are needed. Exercise improves physical fitness and may also improve lung function and quality of life in people with CF [5]. If exercise also aids mucus clearance, it would reduce treatment time, as exercise could substitute airway clearance interventions, while gaining the other known benefits of exercise.

Airway clearance or physiotherapy techniques aim to improve mucus clearance by the following mechanisms: altering airflow (increasing the peak expiratory flow and creating an expiratory airflow bias, with the ratio of peak expiratory to peak inspiratory flow, PEF:PIF > 1.10); [6, 7] improving the physical properties of the mucus; [8] potentially increasing airway surface hydration; [9–13] and coughing [14].

Treadmill exercise improves mucus clearance mechanisms in CF by increasing PEF and reducing sputum mechanical impedance [15]. Physiotherapy with a device creating oscillating positive expiratory pressure, the Flutter®, is an established form of airway clearance in CF and is equally effective to other airway clearance techniques [16]. The Flutter® improves mucus clearance mechanisms in CF by increasing PEF and creating an expiratory airflow bias, [17] as well as reducing sputum mechanical impedance [18]. Exercise and Flutter®, however, have not been compared. Therefore, the aim of this study was to determine the effects of treadmill exercise and Flutter® therapy, compared to resting breathing (control), on respiratory flow (including airflow bias), sputum properties and subjective responses in adults with CF.

## Methods

### Participants

Participants were recruited from the Adult CF Clinic at Royal Prince Alfred Hospital, Sydney, Australia. Patients were eligible for inclusion if they were at least 17 years old, had a confirmed diagnosis of CF (genetic testing and/or previous positive sweat test results) and their treating physician deemed them to be clinically stable [19]. Patients were excluded if they had received a lung transplant, were infected with *Burkholderia cepacia* complex or were pregnant. Potential participants were volunteers or personally approached by one of the researchers (TJD) at either a routine clinic visit or at the end of a hospital admission. Research procedures were approved by the Sydney South West Area Health Service Ethics Committee (Protocol X08-0175) and participants provided written informed consent prior to trial enrolment.

### Study design

**Fig. 1:**
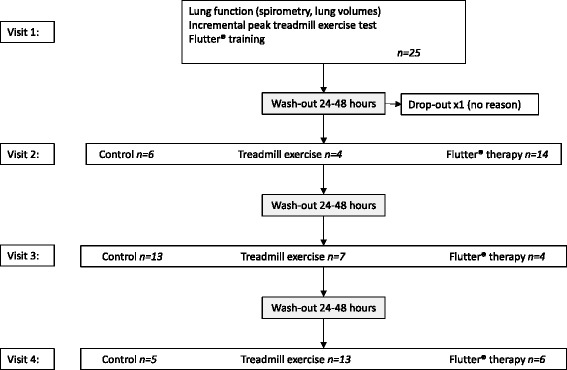
Participant flow during the trial

The trial was a randomised, cross-over design, registered with the Australian and New Zealand Clinical Trials Registry (#ACTRN12609000168257). The study involved four visits (Fig. 1). On Visit 1, participants’ spirometry and lung volumes (via body plethysmograpy) (VMax229, SensorMedics, Yorba Linda, USA) were measured according to the American Thoracic Society/European Respiratory Society guidelines [20, 21]. Participants then completed an incremental peak treadmill exercise test, according to a modified Balke protocol, [22] with breath-by-breath measurement of ventilatory and metabolic variables (VMax229 system) and pulse oximetry (Radical^TM^, Masimo, Irvine, USA). All exercise tests were classified as maximal effort according to the criteria outlined in the CF exercise testing guidelines [23]. Participants were taught to use the Flutter® device (Flutter VRP1 valve®; Axcan Scandipharm Inc., Birmingham, USA) by a senior physiotherapist. If participants were using the Flutter® on a regular basis, any corrections to their technique were made if necessary. After completion of all study procedures on Visit 1, participants were randomised to the order of interventions for the following three sessions (Visits 2, 3 and 4). Intervention order was determined by computer-generated randomisation (with a random integer generator on www.random.org). Randomisation was performed by a person not involved in the interventions on Visits 2, 3 and 4 and stored in sealed, sequentially numbered, opaque envelopes.

**Fig. 2:**
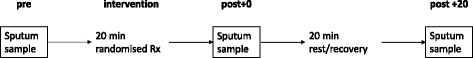
Data collection procedures on Visits 2, 3 and 4. Participants completed visual analogue scores for subjective sense of chest congestion and ease of sputum expectoration with each sputum sample. A sputum sample was also collected five minutes after the intervention (post + 5) if spontaneously expectorated (i.e. it was not requested from participants). Respiratory flow data were collected during the 20 min treatment and coughs were counted during the 20 min treatment and rest/recovery periods

On Visits 2, 3 and 4, participants completed the three-day, randomised, cross-over study, according to the data collection procedures in Fig. 2. Visits 2, 3 and 4 were scheduled at the same time in the morning within a one-week period (during which medication, airway clearance and exercise regimens were unchanged). Participants were also asked to withhold routine mucolytic therapy, airway clearance and exercise on the morning of a trial visit. On each Visit 2, 3 and 4, sputum samples were collected immediately before (pre) and after (post + 0) a 20-min intervention, and after a further 20 min of resting breathing/recovery (post + 20). If participants spontaneously expectorated a sputum sample in the five minutes following the intervention (i.e. they were not requested to do so), this was also collected (post + 5). The three interventions were resting breathing (control), constant-load treadmill exercise and Flutter® plus the forced expiratory technique (FET), [24] from now on referred to as “Flutter® therapy”.

#### Treatment interventions

For the control intervention, participants sat quietly for 20 min. For the exercise intervention, participants exercised on the treadmill for 20 min at a constant work rate equivalent to 60% of the peak oxygen consumption (VO_2_) achieved in the incremental peak treadmill test on Visit 1. This intensity and duration were chosen to replicate a typical prescription used for exercise training [25]. The Flutter® therapy intervention consisted of breathing through the Flutter® for 15 breaths, followed by relaxed and deep breathing, huffing and coughing, according to the FET [24]. This cycle was repeated six times. The Flutter® angle/inclination was chosen for each participant that maximised the sensation of vibrations within the lungs [26] and held in a constant position with a clamp during the intervention. The Flutter® angle was measured with an inclinometer.

### Measurements

#### Respiratory flow

During each 20-min intervention, respiratory flow was measured with a heated pneumotachograph (Hans Rudolf model 3813, Hans Rudolf Inc., Kansas City, USA), calibrated on each occasion, where scaling factors were pre-determined by a rotameter (Model 2000 Fisher Controls, Croydon, England). The Flutter® was attached to the expiratory port of a two-way non-rebreathing valve (2700 series, Hans Rudolf Inc.) in order to collect inspiratory and expiratory flow (i.e. the participant inspired through the pneumotachograph and expired through the pneumotachograph and Flutter®). Data were collected at 125 Hz and flow signals were later analysed by a blinded assessor using custom-made software (PhysioDAQxs v3.0 and Breathalyser v1.0, University of Sydney, Australia) to determine PEF and airflow bias (PEF:PIF) for all interventions, and oscillation frequency during the Flutter® intervention. For the Flutter® intervention, respiratory flow was measured only whilst participants breathed in and out through the Flutter® (i.e. not during the FET component of the intervention).

#### Sputum properties

Sputum samples were manually separated from saliva and stored in 1.2 mL tubes in a −80 °C freezer. The storage tubes were coded, to ensure de-identification at later analysis when measured by a blinded assessor. Sputum analysis procedures were followed as reported previously [15, 27, 28]. The sputum solids content percentage, from which inferences of airway hydration are made, was estimated by measuring the weight of a 50 μL aliquot of sputum before and after lyophilisation to dryness for 24 h using a freeze dryer (Kinetics, Stone Ridge, USA). Sputum elasticity (dynamic G´) and viscosity (dynamic G´´) were measured using a 20 μL aliquot of sputum and a controlled stress rheometer with geometry 20 mm, 0.5° aluminium cone and plate over the frequency of 1–100 rad/s (AR2000, TA Instruments, New Castle, USA). The results were reported as sputum mechanical impedance (G*), also known as rigidity factor, which is the vector sum of viscosity and elasticity. Sputum mechanical impedance values at 1 rad/s represent sputum properties during resting breathing and mucociliary clearance, values at 100 rad/s represent those during cough and cough clearance.

#### Cough

All coughs (spontaneous and those directed, according to the FET) were manually counted during each 20-min intervention and recovery period.

#### Subjective responses

For each requested sputum sample, participants recorded on a 10 cm visual analogue scale the subjective sense of chest congestion (0 = very congested, 10 = very clear) and ease of expectoration (0 = very difficult to expectorate, 10 = very easy to expectorate). The visual analogue scales were later measured by an assessor blinded to the intervention.

### Statistical analyses

Repeated measures ANOVA were performed to compare differences between the interventions in subjective responses and sputum properties data. Paired t-tests were used to compare respiratory flow between the interventions. Wilcoxon signed rank tests were used to determine differences between the interventions in the number of coughs, as these data were not normally distributed. Statistical significance was set at *p* < 0.05.

The difference in sputum mechanical impedance between interventions was the primary outcome measure. Data from our previous study showed that 20 participants would be required to provide 80% power to detect the anticipated between group differences as significant for three of the four measures of sputum mechanical impedance (alpha 0.05) [15]. We sought to recruit 25 participants to allow for a 20% dropout and increase precision around our estimates.

## Results

Twenty-five adults with mild to severe CF lung disease were recruited and 24 completed the study (one participant withdrew after Visit 1 without giving a reason). Participant baseline characteristics are presented in Tables 1 and 2 [29–32]. Routine mucolytic therapy was: hypertonic saline only for 6 participants; rhDNase only for 9 participants; both hypertonic saline and rhDNase for 7 participants. No participant used mannitol and 2 participants did not use any mucolytic medication. Twenty-one of the 24 participants exercised regularly when well and 22 performed some form of airway clearance routinely (3 only exercised; 1 performed established airway clearance only and 18 performed a combination of exercise and established airway clearance techniques, including 2 who performed Flutter® therapy on a regular basis (see Additional file 1 for full details).

**Table 1:** Participant characteristics

	Mean ± SD	Range
Age (yr)	30 ± 8	19–48
Sex (F : M)	9 : 15	
BMI (kg/m^2^)	21.0 ± 2.2	17.1–26.2
FEV_1_ (L)	1.81 ± 0.72	0.90–3.40
FEV_1_ (predicted %)	51 ± 18	28–86
FVC (predicted %)	71 ± 14	46–98
RV/TLC (%)	40 ± 10	24–57
Treadmill peak VO_2_ (mL/kg/min)	30.6 ± 7.8	18.9–50.5
Treadmill peak VO_2_ (predicted %)	82 ± 19	48–127

Mean ± standard deviation and range of participant baseline characteristics for the 24 participants who completed the study. Forced expiratory volume in 1 s (FEV_1_), forced vital capacity (FVC) [30] and treadmill peak VO_2_ [31, 32] expressed as a percentage of predicted values. Residual volume (RV) divided by total lung capacity (TLC) reflects the degree of air trapping

**Table 2:** Baseline sputum properties and subjective reports

	Mean ± SD	Range
Sputum solids content (%)	6.4 ± 2.6	1.6–13.3
Sputum mechanical impedance (G*) at 1 rad/s (Pa)	21.0 ± 15.9	5.7–59.1
Sputum mechanical impedance (G*) at 100 rad/s (Pa)	174.8 ± 76.7	84.1–396.7
Sense of chest congestion (cm)	5.5 ± 2.4	0.5–9.8
Ease of expectoration (cm)	4.9 ± 2.5	0.1–10.0

Mean ± standard deviation and range of sputum properties and subjective reports for the first sputum sample collected from the 24 participants who completed the study. Sputum mechanical impedance (G*, the vector sum of sputum viscosity and elasticity). Subjective sense of chest congestion (0 = very congested, 10 = very clear) and ease of expectoration (0 = very difficult to expectorate, 10 = very easy to expectorate) scored by participant on a 10 cm visual analogue scale

All participants were able to spontaneously expectorate a sputum sample at each requested time point. There were no significant differences in pre-intervention sputum properties or subjective sense of chest congestion and ease of expectoration on Visits 2, 3 and 4, and no carry-over or order effect between interventions was detected (Additional file 1).

### Treatment descriptors

Pulse rate, oxygen saturation and treatment descriptors (work rate and perceived intensity during treadmill exercise; [33, 34] Flutter® angle, oscillation frequency and average expiratory pressure) for the 20-min interventions are presented in Table 3. Treadmill exercise was moderate intensity for breathlessness and perceived exertion. All treatments were well-tolerated with no adverse events.

**Table 3:** Treatment descriptors

	PR (bpm)	SpO_2_ (%)	Treatment descriptors
Control	81 ± 14	96 ± 3	resting breathing
Treadmill	129 ± 18	96 ± 3	5.4 km/h ± 0.7 at 3% incline ± 3, dyspnoea 3 ± 1, RPE 3 ± 2
Flutter®	84 ± 10	97 ± 2	7.3° ± 3.6 at 17.5 Hz ± 1.7, 31 cmH_2_O ± 10

Data are presented as mean ± standard deviation for group values of the pulse rate (PR) and oxygen saturation (SpO_2_), and treatment descriptors (treadmill speed and incline, modified Borg dyspnoea [34] and modified 0-to-10-point rate of perceived exertion (RPE) [33]; Flutter® angle and oscillation frequency, average expiratory pressure). Treadmill work rate was set at the speed and incline equivalent to 60% of the participant’s peak VO_2_ achieved on Visit 1 of the study. Flutter® angle (positive numbers represent an inclination above the horizontal at 0°) was set at the inclination determined to be the most effective by the senior physiotherapist on Visit 1 of the study (i.e. that maximised the sensation of vibrations within the lungs)

### Mucus clearance mechanisms

#### Respiratory flow

Peak expiratory flow (PEF) was significantly higher during treadmill exercise and Flutter® compared to control (Table 4). Only Flutter® resulted in an expiratory airflow bias (PEF:PIF > 1.10).

**Table 4:** Respiratory flow during the interventions

	PEF (L/s)	PEF:PIF
Control	0.68 ± 0.28	0.85 ± 0.14
Treadmill	1.68* ± 0.51	0.90 ± 0.10
Flutter®	1.53* ± 0.25	1.13* ± 0.37

Data are presented as mean ± standard deviation for group values of peak expiratory flow (PEF) and ratio of peak expiratory to peak inspiratory flow (PEF:PIF). Mean difference and (95% CI): Treadmill v control PEF 1.00 L/s (0.82 to 1.18); Flutter® v control PEF 0.85 L/s (0.69 to 1.01); Flutter® v control PEF:PIF: 0.28 (0.11 to 0.45)

*p < 0.01 compared to control

#### Sputum properties

**Fig. 3:**
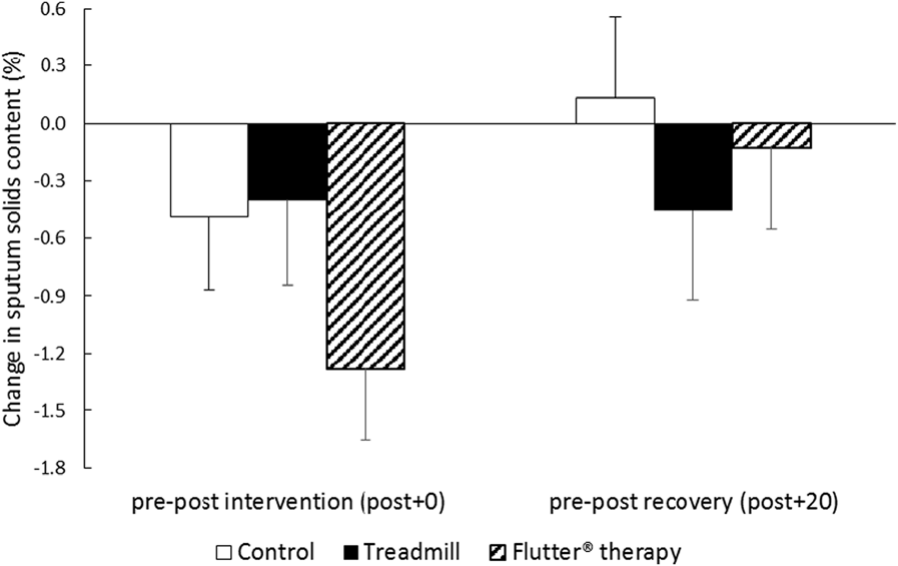
Change in sputum hydration. Measured by sputum solids content, from pre to post intervention (post + 0) and pre to post recovery (post + 20). A negative change represents an improvement in sputum hydration. Results are group mean and SE for the control (*white*), treadmill exercise (*black*) and Flutter® therapy (diagonal lines) interventions

There were no significant differences in sputum water content, measured by sputum percent solids, between any interventions immediately after (post + 0) or after 20-min recovery (post + 20) (Fig. 3). However, for those who spontaneously expectorated a sputum sample in the five minutes following an intervention (post + 5; *n* = 12/15/16 for control/exercise/Flutter® therapy respectively), treadmill exercise resulted in significantly lower sputum percent solids than control (pre-post + 5 mean difference 1.2%, 95% CI 0.4 to 1.9) and a trend for lower sputum percent solids compared to Flutter® therapy (pre-post + 5 mean difference 1.1%, 95% CI −0.1 to 2.3).

**Fig. 4:**
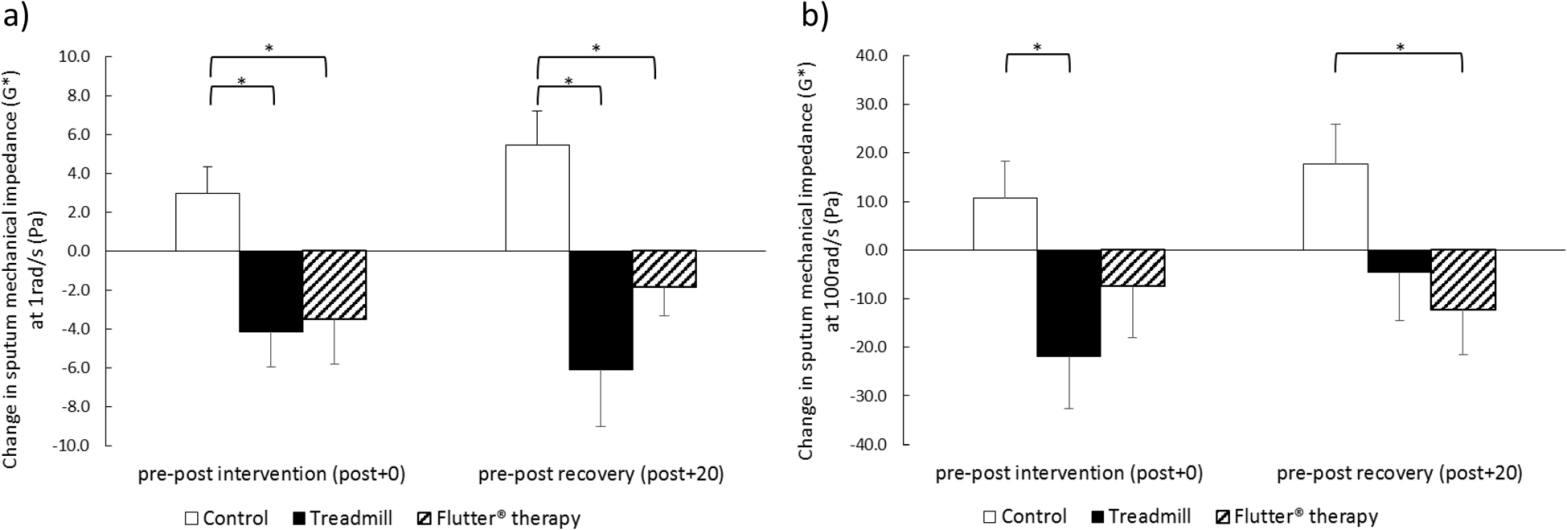
Change in sputum mechanical impedance (G* vector sum of sputum viscosity and elasticity) at (**a**) 1 rad/s and (**b**) 100 rad/s. Measured pre to post intervention (post + 0) and pre to post recovery (post + 20). A negative change represents an improvement in sputum mechanical impedance. Results are group mean and SE for the control (*white*), treadmill exercise (*black*) and Flutter® therapy (diagonal lines) interventions. *p < 0.03

Treadmill exercise resulted in significant reductions in sputum mechanical impedance compared to control both immediately following the intervention (pre-post + 0 mean difference at 1 rad/s 7.1 Pa, 95% CI 1.9 to 12.3; at 100 rad/s 32.5 Pa, 95% CI 5.5 to 59.6) and after 20-min recovery (pre-post + 20 mean difference at 1 rad/s 11.5 Pa, 95% CI 4.0 to 19.1) (Fig. 4). Flutter® therapy resulted in significant reductions in sputum mechanical impedance both immediately following the intervention (pre-post + 0 mean difference 6.4 Pa, 95% CI 0.7 to 12.2) and after 20-min recovery (pre-post + 20 mean difference at 1 rad/s 7.3 Pa, 95% CI 3.3 to 11.2; at 100 rad/s 29.9 Pa, 95% CI 29.9 Pa, 9.0 to 50.9). There were no significant differences in sputum mechanical impedance following treadmill exercise compared to Flutter® therapy.

#### Cough

There were significantly more coughs during treadmill exercise and Flutter® therapy compared to control, and during Flutter® therapy compared to treadmill exercise (Table 5). Note that participants were instructed to cough 18 times during the FET in the Flutter® therapy intervention. There were no differences between interventions in the number of spontaneous coughs during the 20-min recovery.

**Table 5:** Coughs during and following the interventions

	Coughs during intervention	Coughs during recovery
Control	2 (0–5)	1 (0–3)
Treadmill	4* (1–9)	2 (1–5)
Flutter® therapy	24* (18–34)	2 (1–4)

Data are presented as median (interquartile range) for group values of the number of coughs during the 20-min intervention and 20-min resting breathing/recovery period. NB. Participants were instructed to cough 18 times during the Flutter® therapy intervention

*p < 0.01 compared to control

### Subjective responses

**Fig. 5:**
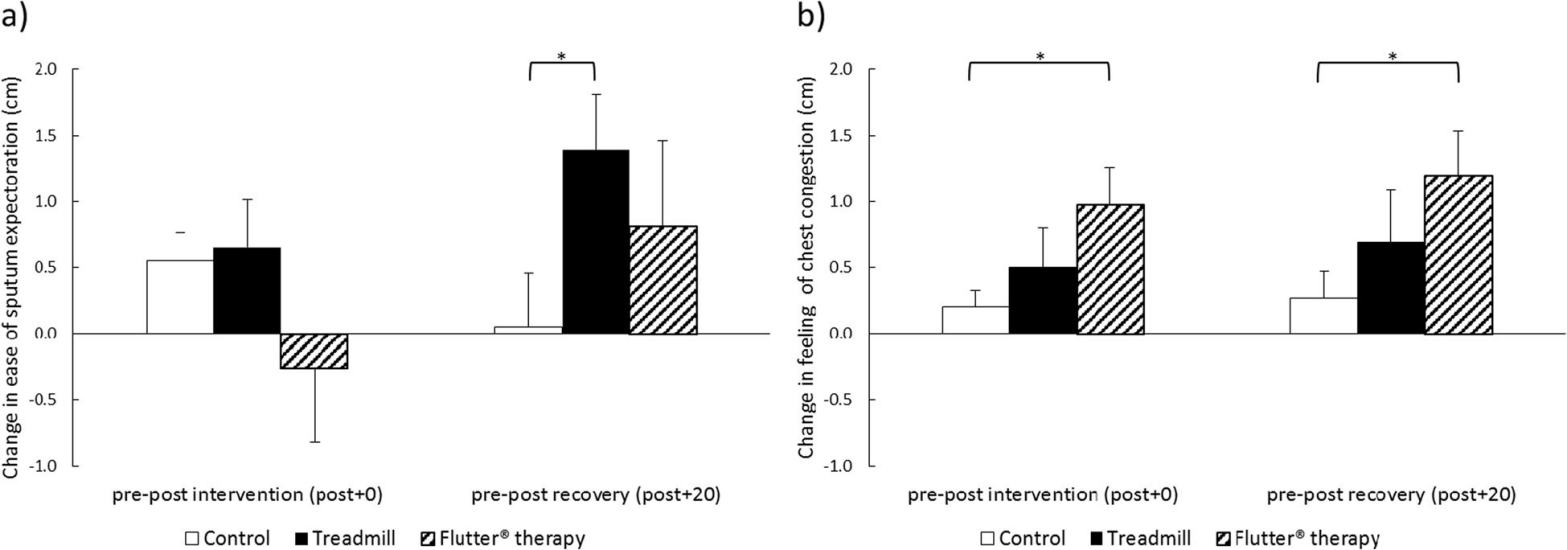
Change in subjective (**a**) ease of sputum expectoration and (**b**) feeling of chest congestion. Measured on a 10 cm visual analogue scale, from pre to post intervention (post + 0) and pre to post recovery (post + 20). A positive change represents an improvement in ease of sputum expectoration and chest congestion. Results are group mean and SE for the control (*white*), treadmill exercise (*black*) and Flutter® therapy (diagonal lines) interventions. *p < 0.02

Treadmill exercise significantly improved subjective ease of expectoration compared to control after 20-min recovery (pre-post + 20 mean difference 1.3 cm, 95% CI 0.3 to 2.3) (Fig. 5a). There were no significant differences in ease of expectoration following Flutter® therapy compared to control or Flutter® therapy compared to treadmill exercise.

There were no significant differences in subjective sense of chest congestion following treadmill exercise compared to control or treadmill exercise compared to Flutter® therapy (Fig. 5b). Flutter® therapy significantly improved subjective sense of chest congestion compared to control both immediately post intervention (pre-post + 0 mean difference 0.8 cm, 95% CI 0.1 to 1.4) and after 20-min recovery (pre-post + 20 mean difference 0.9 cm, 95% CI 0.2 to 1.7).

## Discussion

The primary purpose of this study was to compare treadmill exercise and Flutter® therapy on mucus clearance mechanisms in CF. The main findings were that both treadmill exercise and Flutter® resulted in similar significant increases in PEF, but only Flutter® created an expiratory airflow bias. In addition both treadmill exercise and Flutter® therapy resulted in similar significant reductions in sputum mechanical impedance, but only treadmill exercise caused a transient increase in sputum hydration.

The PEF and airflow bias measured during treadmill exercise was similar to that reported by our group previously [15]. The PEF and oscillation frequency measured during Flutter were higher than previously reported by our group (1.53 L/s v 1.13 L/s and 17.5 Hz v 11.3 Hz respectively), yet the airflow bias was similar (1.13 v 1.15), [17] and above the 1.10 threshold proposed to augment annular flow of mucus towards the oropharynx [6]. The higher PEF and oscillation frequency with Flutter® in this study compared to our earlier work may be explained by the Flutter® position. In the earlier study the Flutter® was used in the horizontal position for all participants, [17] however in the current study the Flutter® inclination was individually determined (with an average angle 7.3° above the horizontal). Holding the Flutter® at higher inclinations results in higher oscillations [35, 36].

The reductions in sputum mechanical impedance following treadmill exercise were similar to those reported previously by our group [15]. Different techniques to measure sputum viscosity and elasticity prevented comparing the changes following Flutter® therapy in this study to those reported by other researchers [18]. Our study found no significant difference between treadmill exercise and Flutter® therapy in the reductions in sputum mechanical impedance, suggesting that the combined effects of shearing forces and airway oscillations with the two interventions were similar.

There was no change in sputum hydration immediately following treadmill exercise or after 20 min of recovery, similar to our previous study [15]. However, we found a significant reduction in sputum solids content in the five minutes following treadmill exercise but not after Flutter® therapy. Previously researchers have shown an inhibition of sodium conductance channels [11, 12, 37] and altered ion regulation with submaximal cycle exercise in adults with CF [13], suggesting improved airway hydration or airway surface liquid, however these changes only lasted for four minutes after ceasing exercise [37]. Our study provides some evidence to support the proposed increase in mucus water content with exercise in CF [10–12]. The 1.2% reduction in sputum solids content that we observed is likely to be clinically significant as it is similar to that achieved with mannitol in people with CF, [28] which results in significant improvements in mucus clearance [38] and lung function in the long term [39].

Consistent with the improved changes in sputum properties, participants reported significant improvements in ease of expectoration following treadmill exercise but not following Flutter® therapy. Alternately, participants reported significant improvements in subjective sense of chest congestion following Flutter® therapy but not following treadmill exercise. We did not measure the amount of sputum expectorated, as this would have interfered with sputum rheology and solids content measurements. Perhaps treadmill exercise facilitated sputum expectoration (due to increased PEF and reduced sputum mechanical impedance), but participants did not spontaneously expectorate sufficient sputum to feel less chest congestion. Also, potentially the format of the FET during Flutter® treatment (18 directed coughs in 20 min) increased the amount of sputum expectorated (and hence sensation of less chest congestion), but it was a taxing treatment and so participants did not consider it easier to expectorate.

## Conclusions

A single bout of moderate-intensity treadmill exercise and Flutter® therapy improved mucus clearance mechanisms in adults with CF. Both treatments increased PEF, but only Flutter® created an expiratory airflow bias. Both treatments resulted in similar significant reductions in sputum mechanical impedance, however only treadmill exercise created a significant transient reduction in sputum solids content. It would therefore appear that treadmill exercise and Flutter® therapy are equally effective in augmenting mucus clearance mechanisms in adults with CF. Physiological or mechanistic findings on their own, however, are insufficient to implement changes to clinical practice. Studies that directly measure mucus and mucociliary clearance or longer term studies with clinically important outcomes (such as exacerbation frequency, antibiotic use, quality of life and lung function) are required to ascertain the relative merit of these interventions and to determine if people with CF can use exercise alone as an adequate form of airway clearance therapy.

## Additional file

Additional file 1. Excel spreadsheet includes participant characteristics, treatment descriptors, and the results of interventions (respiratory flow and cough, sputum properties and subjective responses. (XLSX 65 kb)

## Abbreviations

CF: cystic fibrosis
FET: forced expiratory technique
FEV_1_: forced expiratory volume in 1 s
G*: sputum mechanical impedance
PEF: peak expiratory flow
PIF: peak inspiratory flow
RPE: rate of perceived exertion
RV: residual volume
Rx: treatment
TLC: total lung capacity
VO_2_: oxygen consumption
